# Neuropeptide W regulates proliferation and differentiation of ATDC5: Possible involvement of GPR7 activation, PKA and PKC‐dependent signalling cascades

**DOI:** 10.1111/jcmm.14118

**Published:** 2019-01-04

**Authors:** RiKang Wang, Chaojun Zheng, Wenyu Jiang, Xinshu Xie, Rifang Liao, Guangqian Zhou

**Affiliations:** ^1^ Shenzhen Key Laboratory for Anti‐ageing and Regenerative Medicine, Guangdong Key Laboratory for Genome Stability and Disease Prevention, Department of Medical Cell Biology and Genetics Shenzhen University Health Science Center Shenzhen China; ^2^ National Pharmaceutical Engineering Center for Solid Preparation in Chinese Herbal Medicine Jiangxi University of Traditional Chinese Medicine Nanchang China; ^3^ Department of pharmacy Sun Yat‐sen Memorial Hospital, Sun Yat‐sen University Guangzhou P.R. China

**Keywords:** ATDC5, chondrocytes, chondrogenic differentiation, GPR7, neuropeptides W, proliferation

## Abstract

Various neuropeptides related to the energy equilibrium affect bone growth in humans and animals. Neuropeptides W (NPW) are identical in the internal ligands of the two G‐protein receptors (GPRs) included in subtypes 7 and 8. Neuropeptides W inhibits proliferation in the cultivated rat calvarial osteoblast‐like (ROB) cells. This study examines the expression of NPW and GPR7 in murine chondrocyte and their function. An immunohistochemical analysis showed that NPW and GPR7 were expressed in the proliferative chondrocytes of the growth plates in the hind limbs of mice. The NPW mRNA quickly elevated in the early differentiation (7‐14 days) of ATDC5 cells, while NPW and GPR7 mRNA were reduced during the late stage (14‐21 days) of differentiation. Neuropeptide W‐23 (NPW‐23) promoted the proliferation of ATDC5 cells, which was attenuated by inhibiting the GPR7, protein kinase A (PKA), protein kinase C (PKC) and ERK1/2 pathways. Neuropeptide W‐23 enhanced the early cell differentiation, as evaluated by collagen type II and the aggrecan gene expression, which was unaffected by inhibiting the ERK1/2 pathway, but significantly decreased by inhibiting the PKA, PKC and p38 MAPK pathways. In contrast, NPW‐23 was not involved in the terminal differentiation of the chondrocytes, as evaluated by the mineralization of the chondrocytes and the activity of the alkaline phosphatase. Neuropeptides W stimulated the PKA, PKC, p38 MAPK and ERK1/2 activities in a dose‐ and time‐dependent manner in the ATDC5 cells. These results show that NPW promotes the proliferation and early differentiation of murine chondrocyte via GPR7 activation, as well as PKA and PKC‐dependent signalling cascades, which may be involved in endochondral bone formation.

## 1 INTRODUCTION

Neuropeptides W (NPW) and Neuropeptides B (NPB) have been identified as endogenetic ligands of G‐protein receptors (GPR) 7 and 8.^1^, ^2^ Both GPR7 and GPR8 are expressed in humans, but GPR8 is absent in rodents.^3^ Neuropeptides W, NPB and their receptors are mainly expressed in the central and periphery issues, which are involved in many physiological processes, including inflammatory pain, energy homeostasis, cardiovascular functions, immune system, stress and the neuroendocrine and respiratory systems.^4^, ^5^, ^6^ Previous studies have detected NPW/NPB mRNA, including bone marrow, femur and costal cartilagein, in humans, rats, pigs and chickens.^1^, ^7^, ^8^ The effect of G protein activation was mediated by protein kinase A (PKA), protein kinase C (PKC) and the mitogen‐activated protein kinases (MAPKs) cascades reaction.^9^, ^10^ The down‐regulation or inhibition of PKA and PKC blocks chondrogenesis.^11^, ^12^ The proliferation and differentiation of chondrocytes are regulated by PKC‐mediated p38 MAPK and the ERK1/2 signalling pathway.^13^ The PKA and PKC cascades are relevant to the secret agogue effect of NPW and NPB in human adrenocortical cells.^9^ Neuropeptides W stimulates the proliferation of NCI‐H295 cells, which are derived from human adrenocortical carcinoma by exerting the ERK1/2 pathway,^14^ which is considered a crucial growth factor in rat adrenocortical cells.^15^

Neuropeptides and their receptors are expressed in bone tissue and are involved in bone development in humans and animals.^16^, ^17^, ^18^ Neuropeptides W, NPB and their receptors are expressed and inhibited proliferative activity in cultured rat calvarial osteoblast‐like (ROB) cells.^19^ However, little is known about whether NPW/B can regulate endochondral bone formation. The role of NPW/B in the regulation of the chondrocyte function has not been characterized so far. Therefore, we used immunohistochemical analyses to assess the expression of NPW and it's receptor in the growth plates of mice. We also identify the role of NPW and GPR7 in chondrocyte using an excellent in vitro model cell line called ATDC5 for chondrocyte proliferation and differentiation. The ATDC5 cell line is derived from AT805 teratocarcinoma cells and is characterized as a chondrogenic cell line that is capable of differentiating into chondrocytes.^20^, ^21^ The molecular analysis of early‐ and late‐phase differentiation markers of chondrocytes in vivo can also be mimicked by ATDC5 cells in vitro.

## 2 MATERIALS AND METHODS

### 2.1 Animals and reagents

Kunming mice (male, 25‐35 g, 7‐8‐week‐old) were purchased from the Laboratory Animal Centre at the Jiangxi University of Traditional Chinese Medicine. Neuropeptide W‐23 (NPW‐23) and the EIA Kit of NPW‐23 (Rat, Mouse) were purchased from Phoenix Biotech (Beijing, China). H‐89, Chelerythrine (Chele), PD‐98059, SB‐203580 and JNK inhibitor were purchased from Calbiochem (La Jolla, CA, USA). The CYM 50769 (GPR7 antagonist) was purchased from Tocris Bioscience. The anti‐NPW antibody and anti‐GPR7 antibody were purchased from Absin Bioscience Inc. (Shanghai, China);and the anti‐phospho‐p38 (Thr180/Tyr182) antibody, anti‐Phospho‐ERK1/2 (Thr202/Tyr204) antibody, anti‐Phospho‐PKA (Ser/Thr) antibody, anti‐p38 antibody, anti‐PKA antibody, anti‐PKC antibody and anti‐phospho‐PKCδ (Thr505) antibody were obtained from Cell Signaling Technology (Woburn, MA, USA). The siRNAs for the GPR7 were designed and synthesized by Gen Pharma Co., Ltd. (Shanghai, China). Finally, the primers for the NPW, GPR7, p21, aggrecan, Sox‐9, Coll II, Coll X, Runx2 and ribosomal protein L19 (RPL‐19) were designed and synthesized by Invitrogen Co. (Guangzhou, China).

### 2.2 Immunohistochemistry analysis

Slides were processed as described above for the immunohistochemical analysis.^22^ The tibiofemoral joints were fixed briefly in 10% paraformaldehyde for 24 hours after the knee samples were harvested. The fixed femoral condyles were decalcified in 10% EDTA for 7 days at 37°C. After graded ethanol dehydration and dimethylbenzene vitrification, the femoral condyles were embedded in paraffin and sectioned at 5 μm thickness. The sections were then incubated with primary antibodies against anti‐NPW and anti‐GPR7 antibodies overnight at 4°C. The biotinylated secondary antibody and streptavidin peroxidase solution were then used to visualize the sections.

### 2.3 Cell culture

ATDC5 cells were cultured in 1:1 mixed DMEM and Ham'sF‐12 media containing 10% fetal bovine serum, 100 units/mL penicillin and 100 μg/mL streptomycin (Sigma‐Aldrich, St. Louis, MO). The ATDC5 cells were cultured at 37°C in a humidified atmosphere of 5% CO_2_ in air. Insulin, transferin and selenite (ITS, Sigma‐Aldrich) were added to the medium to induce ATDC5 cell differentiation in the culture.

### 2.4 Small interfering RNA transfections

The ATDC5 cells were transfected with small interfering RNA (siRNA) of GPR7 or negative control siRNA using a Lipofectamine 2000 reagent according to the manufacturer's protocols. The siRNA of GPR7 used in the experiment was designed and synthesized by GenPharma Co., Ltd. (Shanghai, China), and the sequences were as follows: sense 5ʹ‐CGCAUGAAGACUGUUACCATT‐3ʹ, antisense: 5ʹ‐UGGUAACAGUCUUCAUGCGTT‐3ʹ. RT‐PCR was used to verify the GPR7 knockout efficiency.

### 2.5 RT‐PCR analysis

The total RNA was extracted using TRNzol Universal Reagent (Tiangen Biotech, Beijing, China) according to the manufacturer's instructions. The first strand of cDNA was produced using the First‐Strand cDNA Synthesis Kit (Thermo Scientific, USA). The reaction conditions for the PCR and gene‐specific primers (Table 1) were the same as previously above,^23^ and the cDNA was initially pre‐denaturated at 94°C for 10 minutes, followed by 34 cycles of denaturation at 94°C for 30 seconds, annealing at 55°C for 30 seconds, and extension at 72°C for 60 seconds. The PCR products were detected using electrophores is on 1.5% agarose gel stained with ethidium bromide, and the size was compared with the DNA ladder (O'RangeRuler 100‐bp DNA Ladder [MBI Fermentas, Lithuania]). The reverse transcriptase (RT‐PCR) was used as a negative control, and the results were normalized by RPL‐19.

**Table 1:** Primers used for PCR (all primers suitable for mouse cDNA, S, sense; A, antisense)

Gene	Sequence	Accession number
NPW	S 5′‐ACTGCTGCTTCTGCTCTTGC‐3′ A 5′‐GCGTCTCACCGAAGGCTCTA‐3′	NM_001099664
GPR7	S 5′‐CATCTGCGCCCTCTATATCA‐3′ A 5′‐GAAGTAAGAGATGCCGATGACC‐3′	NM_010342
Coll II	S 5′‐AGGGCAACAGCAGGTTCACATAC‐3′ A 5′‐TGTCCACACCAAATTCCTGTTCA‐3′	NM_001844
p21	S 5′‐GGCCCGGAACATCTCAGG‐3′ A 5′‐AAATCTGTCAGGCTGGTCTGC‐3′	NM_007669
Aggrecan	S 5′‐TCTTTGCCACCGGAGA‐3′	L07049
	A 5′‐TTTTTACACGTGAA‐3′	
Sox‐9	S 5′‐GCTGGAAGTCGGAGAGCCGAGA‐3′	NM_011448
	A 5′‐AGAGAACGAAACCGGGGCCAC‐3′	
Coll X	S 5′‐ CTCCTACCACGTGCATGTGAA‐3′ A 5′‐ACTCCCTGAAGCCTGATCCA‐3′	NM_000493
Runx2	S 5′‐ GGTTGTAGCCCTCGGAGAGG‐3′ A 5′‐GCCATGACGGTAACCACAGTC‐3′	NM_001145920

GPR, G‐protein receptor; NPW, neuropeptides W.

### 2.6 Quantitative real‐time PCR

Relative quantification real‐time PCR was performed with SYBR Green PCR Master Mix (Thermo Fisher Scientific, Woolston Warrington, UK) according to the manufacturer's protocols, 2μL of cDNA template, and the gene specific primer sets (Table 1). PCR was carried out in the ABI 7500 system (Applied Biosystems). Relative gene expression was calculated using the 2^–ΔΔCT^ method and RPL‐19 was used as a housekeeping gene for normalization.

### 2.7 Western blotting analysis

The Western blotting analysis was described previously.^24^ Briefly, cells from different experimental conditions were lysed with ice‐cold RIPA lysis buffer. The protein concentration was measured with a BCA protein assay kit according to the manufacturer's instructions. SDS‐PAGE electrophoresis was carried out with the same amounts of lysate protein (20 μg/lane) and 10% polyacrylamide gels and electrophoretically transferred to nitrocellulose membranes. After transfer nitrocellulose blots were first blocked with 3% bovine serum albumin (BSA) in PBST buffer (PBS with 0.01% Tween 20, PH 7.4), and then incubated overnight at 4°C with primary antibodies in PBST containing 1% BSA. Horseradish peroxidase‐coupled secondary antibody was incubated continuously to determine the immunore activity, and enhanced chemiluminescence technique was used to detect the immunore activity.

### 2.8 CCK‐8 assay

ATDC5 cells were seeded in 96‐well plates at the density of 1000 cells per well with 100 μL of culture medium in the 5% (v/v) Fetal Bovine Serum (FBS) culture medium. After adhesion for 24 hours, the medium was changed to serum‐free and NPW‐23 was added to medium with final concentrations ranging from 25 to 400 ng/mL, then the cells were re‐cultured for 24 hours. The cells which were not exposed to NPW‐23 were used as controls. The wells with only culture medium was served as blanks. Afterward, CCK‐8 was added to each well and the plates were incubated at 37°C for additional 3 hours. Then the absorbance of each solution was measured at 450 nm using BIO‐RAD 680 plate reader (Thermo, Walsam, MA, USA). All the experiments were repeated at least three times using triplicate cultures (n = 9).

### 2.9 BrdU cell proliferation assay

Cell proliferation was assessed using BrdU cell proliferation assay kits according to the manufacturer's instructions. Briefly, the cells suspended in the growth medium were seeded in a 96‐well plates with a density of 1 × 10^4^ cells/well and grown overnight at 37°C in a humidified incubator with 5% CO_2_. The second day, the cells were incubated with NPW‐23 (25‐400 ng/mL) for 24 hours. Afterward, the cells were incubated with 20 μL of BrdU for another 12 hours. Fixing solution was then added and incubated for 30 minutes at room temperature. After washing, cells were incubated with a pre‐diluted detector antibody for 1 hour at room temperature. Acid stop solution was added after incubation with HRP‐conjugated secondary antibody for 1 hour at room temperature. The optical density (OD) was then measured at 450 nm using an automated microplate reader.

### 2.10 Alcian blue and Alizarin red staining

The cells were cultured in 12‐well plates at a density of 50 000 cell/well for 4, 7, 14 and 21 days, and were treated with or without indicated concentrations of NPW‐23. Cells were then fixed in 4% (v/v) formalin for 15 minutes, and then stained with Alcian blue 8GX for 30 minutes or with 1% (w/v) Alizarin red for 10 minutes. The stained cells were washed for three times with 1× PBS, then photographed with a scanning camera.

### 2.11 Alkaline phosphatase assay

ATDC5 cells were seeded in 6‐well plates at a density of 100 000 cell/well and then treated with the indicated amounts of NPW‐23. The harvested cells were re‐suspended in lysis buffer and alkaline phosphatase (ALP) activity was measured in a reaction mixture of 0.5 mmol/L *p*‐nitrophenyl phosphate (Sigma‐Aldrich) as the substrate and 0.5 mmol/L MgCl_2_ for 15 minutes at 37°C. *p*‐Nitrophenol concentration was determined at 405 nm. Protein content in cell lysates was measured by BCA protein Kit (Beyotime Biotechnology).

### 2.12 Statistical analysis

All the results were reported as means ± SEM for 3‐5 times. ANOVA was used to analyse the differences between the groups, followed by the TukeyeKramer or Dunnett's multi‐comparison test with pasw Software (SPSS Inc., Chicago, IL, USA). *P* < 0.05 was considered statistically significant.

## 3 RESULTS

### 3.1 Detection of NPWand GPR7 expression in the growth plate

**Figure 1:**
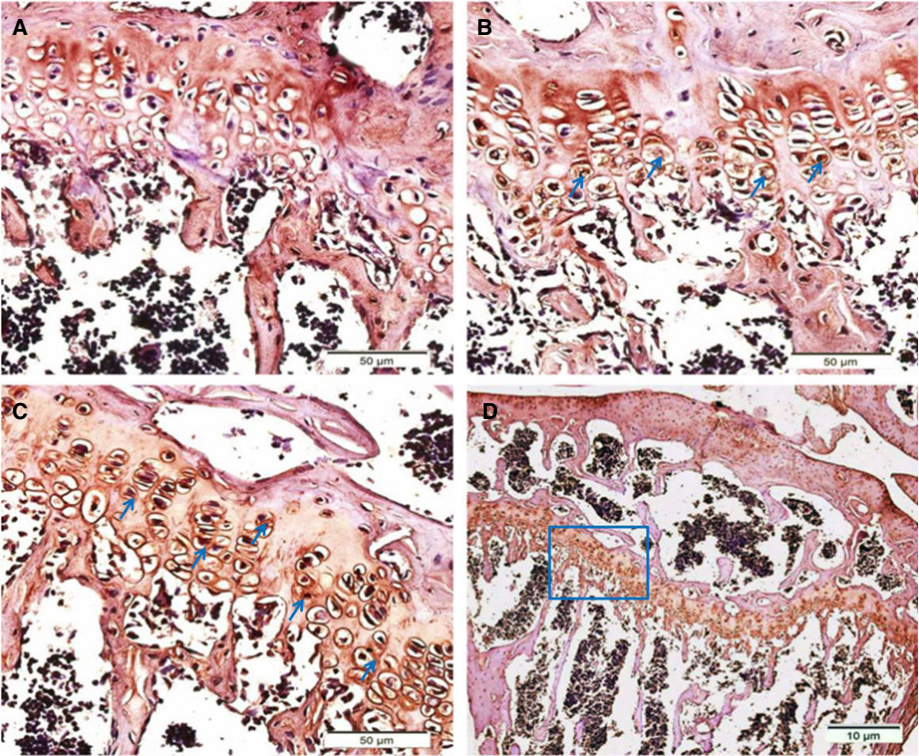
Immunostaining of neuropeptides W (NPW) and GPR7 in the growth plates of mouse hind limb joints. A, No signal was detected when the joints were incubated without primary antibodies. B, Immunostaining results for NPW in joints at high magnification, arrows mark examples of NPW‐positive chondrocytes. Immunostaining results for GPR7 in the joints are shown at high (C) and low (D) magnification, arrows denote examples of GPR7‐positive chondrocytes. D, Box indicates areas enlarged in (C)

An immunohistochemical study of the tibiofemoral joints, which used specific antibodies, detected NPW and GPR7 proteins in the round chondrocytes in the growth plate. As shown in Figure 1, NPW and GPR7 were expressed in the epiphyseal chondrocytes of the mouse limb tissue. NPW (Figure 1B) and GPR7 (Figure 1C) were more strongly expressed in the proliferative chondrocytes of the lower zone of the mouse limb growth plates compared with cells in the upper proliferative and hypertrophic zones.

### 3.2 Expression levels of NPW and GPR7 when differentiating ATDC5 cells

**Figure 2:**
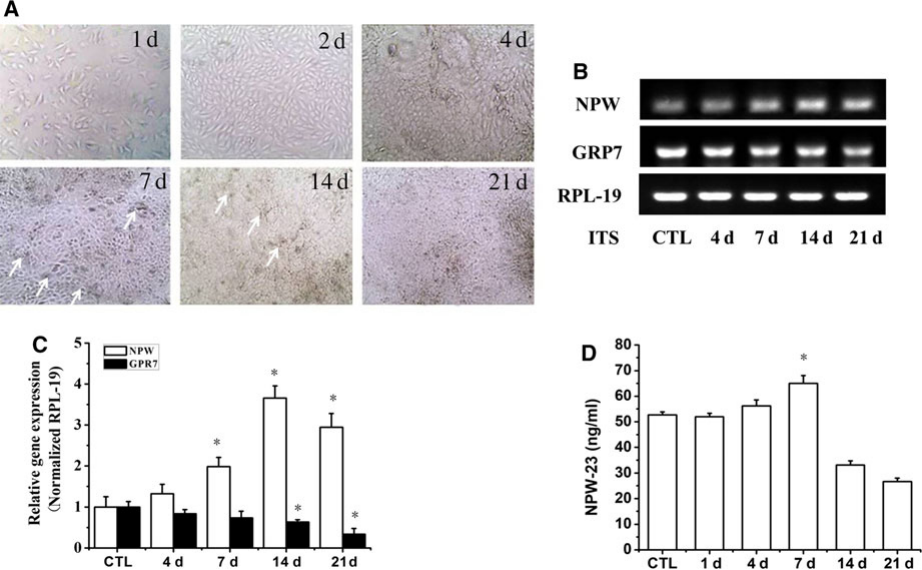
Expression levels of neuropeptides W (NPW) and GPR7 in differentiating ATDC5 cells. ATDC5 cells were cultured in DMEM/F12 medium containing 5% FBS and 1% ITS for 1, 2, 4, 7, 14 and 21 d in a 12‐well plate with a density of 50 000 cells/well. A, Morphological changes were shown by light microscope (10×) image analysis, arrows mark the nodule formation. (B,C) Relative expression of NPW and GPR7 were determined by semi‐quantitative PCR analyses at the indicated periods of ATDC5 differentiation, and RPL‐19 was used as the internal control. D, The concentration of NPW‐23 in the culture medium were analysed at 1, 4, 7, 14 and 21 d after ITS treatment using EIA kit. Data represent as means ± SE from triplicate samples in two independent experiments.*P < 0.05 vs control group

We used a differential medium (containing 1% insulin, transferrin and selenite [ITS]) to stimulate the chondrogenic differentiation of the ATDC5 cells at different times (1‐21 days).Consistent with the previous studies, we observed the condensation stage (day 4) and the nodule formation stage (days 7‐14) in the ATDC5 cells (Figure 2A). We examined the expression levels of GPR7 and NPW‐23 in the cultures of the ATDC5 cells. The RT‐PCR results showed that the mRNA of the NPW expression transiently increased on day 7, and the mRNA peaked on day 14 during differentiation with ITS, while the mRNA of the GPR7 significantly decreased on days 7 and 14 (Figures 2B2, 3, 4, 5, 6C). To determine whether the NPW‐23 secreted from the ATDC5 cells, we assayed the culture medium for the presence of NPW‐23 using an RIA kit. One of the main peaks in the immunoreactive NPW‐23 was detected on day 7 during differentiation with ITS, and the content of the immunoreactive NPW‐23 in the ATDC5 culture medium was 65 ng/mL (Figure 2D).

**Figure 3:**
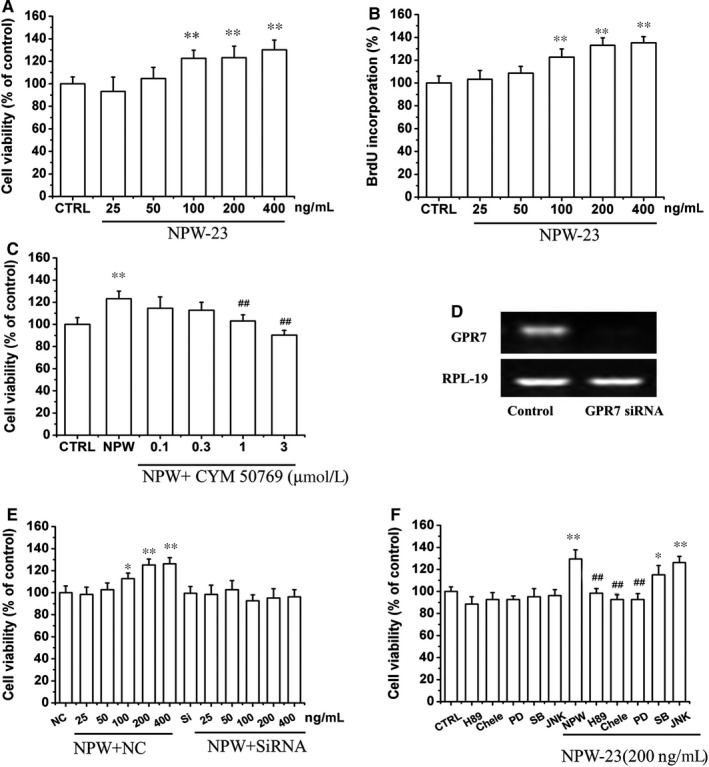
The effect of neuropeptide W‐23 (NPW‐23) on ATDC5 cell proliferation. ATDC5 cells were cultured in 96‐well plates at a density of 25 000 cells/well, and different concentrations of NPW‐23 (25, 50,100, 200, and 400 ng/mL) were used to treat the cells for 24 h. Cell proliferation was determined by CCK‐8 assay (A) and BrdU cell proliferation assay (B), respectively. C, Different concentrations of CYM 50769 for 30 min, ATDC5 cells were exposed with or without 200 ng/mL NPW‐23 for 24 h, and the cell proliferation was measured by CCK‐8. D, ATDC5 cells were transfected GPR7 siRNA for 36 h, and the knockdown efficiency of the GPR7 was detected by RT‐PCR. E, ATDC5 cells with or without GPR7 knockdown were exposed to NPW‐23 for 24 h, and the cell viability was measured by CCK‐8 assay. F, Effects of protein kinase A, protein kinase C and mitogen‐activated protein kinases pathway inhibitors on NPW‐23‐induced proliferation. ATDC5 cells were treated with various pathway inhibitors (H89; Chele; PD98059; SB203580; JNK inhibitors) for 30 min followed by treatment with NPW‐23 for 24 h. CCK‐8 assay was used to determine cell viability. Data is expressed as a percentage of the corresponding control. Results are shown as the mean ± SEM, and represent assays from three independent experiments. *P < 0.05, **P < 0.01 vs control group, ^#^ *P* < 0.05, ^##^ *P* < 0.05 vs NPW‐23 group.

**Figure 4:**
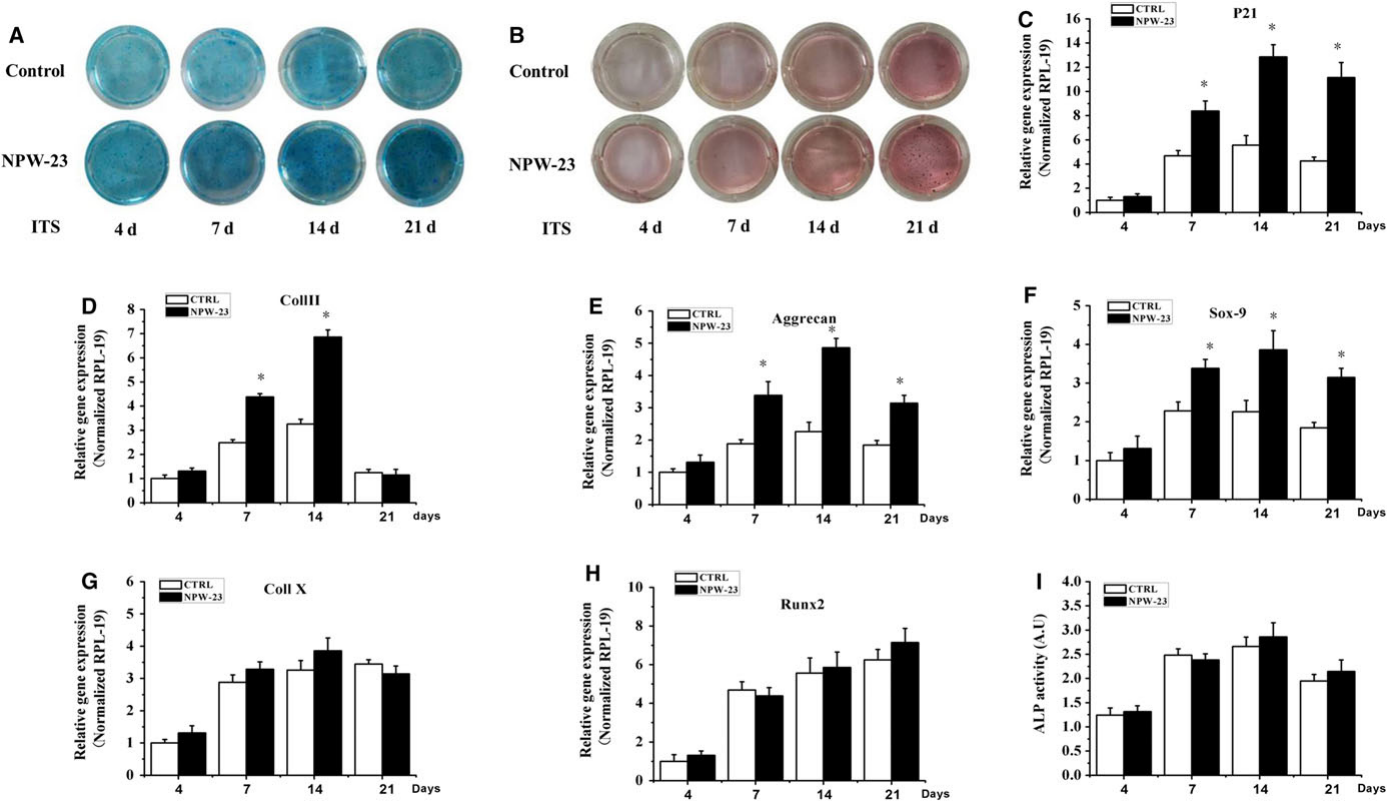
The effect of NPW‐23 on ATDC5 cell differentiation. ATDC5 cells were cultured with DMEM/F12 medium in a 12‐well plate with adensity of 50 000 cells/well. After 12 h, the cells were treated with or without NPW‐23 (200 ng/mL) containing 5% FBS and 1% ITS in DMEM/F12 medium for 4, 7, 14 and 21 d. (A,B) Alcian blue and Alizarin red staining were used to detect the proteoglycan synthesis and mineralization, respectively. (light microscopy, 10× magnification). Effects of NPW‐23 on mRNA levels of (C) p21, (D) Coll II, (E) Aggrecan, (F) Sox‐9, (G) Coll X, and (H) Runx2 in ATDC5 cells were analyzed by real‐time PCR, normalized against RPL‐19 and compared to the control group. I, Alkaline phosphatase (ALP) activity was assessed by ALP assay and described in the methods section of this paper. The data represent triplicate samples in two independent experiments. *P < 0.05 was considered significant

**Figure 5:**
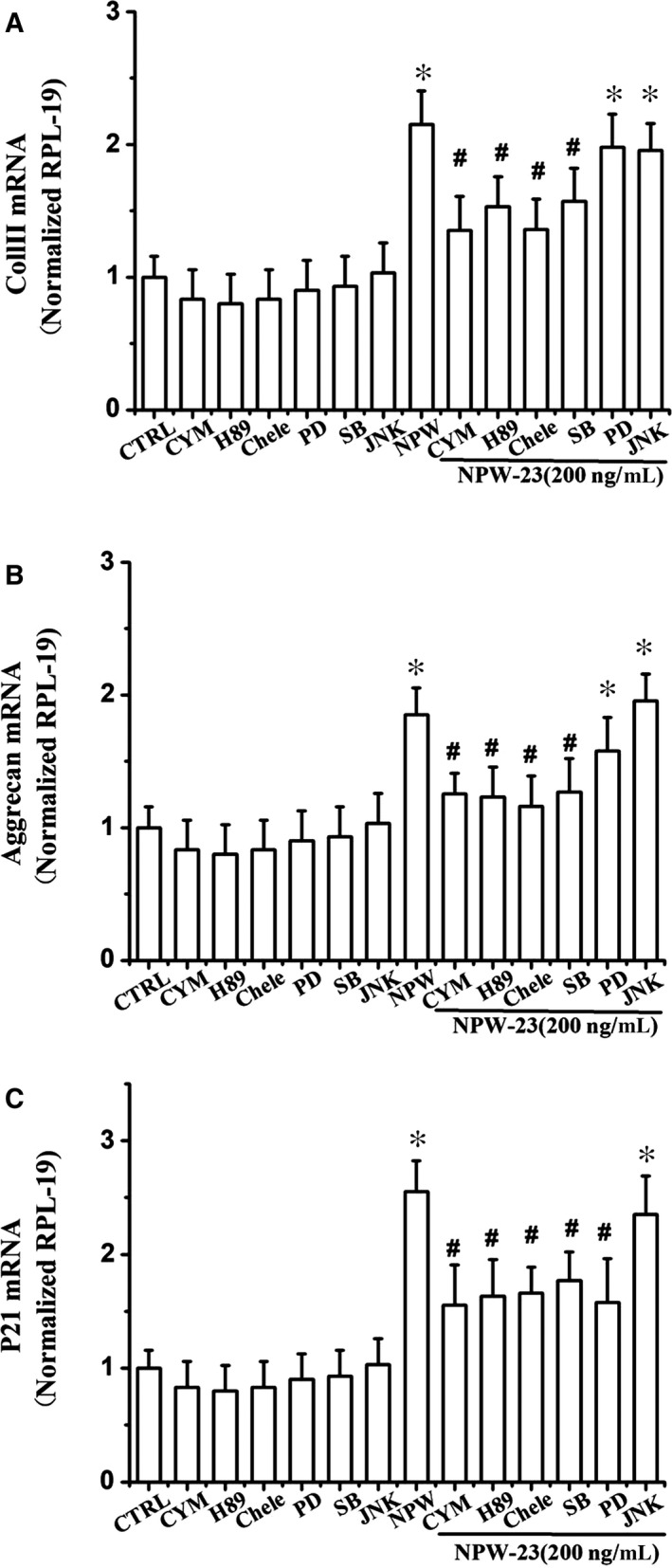
Neuropeptide W‐23 (NPW‐23)‐enhanced differentiation of ATDC5 cells through the protein kinase A, protein kinase C, ERK1/2, and p38 signalling pathways. Cells were cultured in differentiation medium (1% ITS) and incubated with or without NPW‐23 (200 ng/mL). On day 7 of the culture process, the cells were in the presence or absence of specific inhibitors of the different signalling pathways (CYM50769; PD98059; SB203580; Chele; H‐89). After 12 h, the total RNA was extracted and (A) Coll II, (B) aggrecan, and (C) p21 mRNA were detected by quantitative RT‐PCR. Each experiment was repeated at least three times. The data represent triplicate samples in two independent experiments. *P < 0.05 vs control group, ^#^ *P* < 0.05 vs NPW‐23 group.

**Figure 6:**
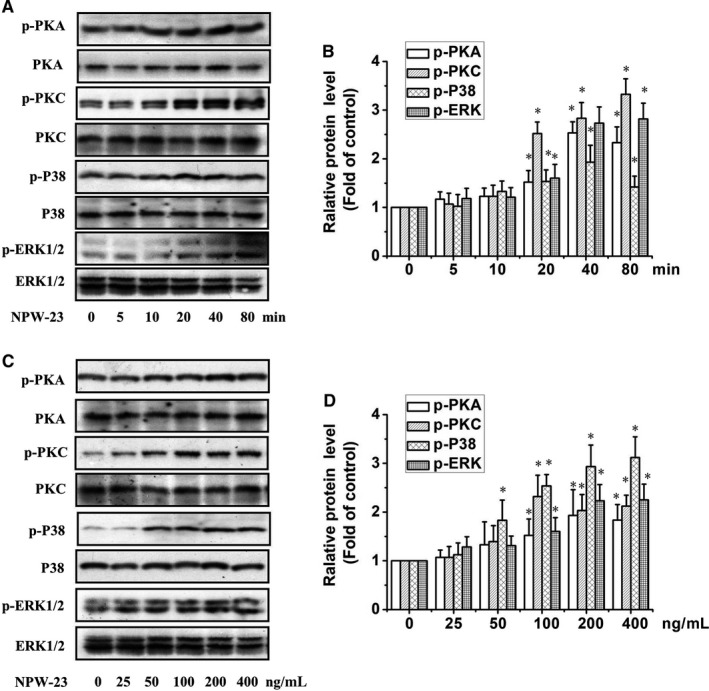
Neuropeptide W‐23 (NPW‐23) time‐ and dose‐dependently increased the phosphorylation level of protein kinase A (PKA) (Ser/Thr), PKCδ (Thr180/Tyr182), p38 (Thr180/Tyr182), and ERK1/2(Thr202/Tyr204) in ATDC5 cells. ATDC5 cells were cultured in differentiated medium (contain 1% ITS) on the day 7, then cells were cultured in serum‐free medium for 12 h, then cells treated with (A) NPW‐23(200 ng/mL) for 10‐80 min; or (C) different concentration of NPW‐23(25‐400 ng/mL) for 40 min. The phosphorylation of PKA, PKC p38 and ERK1/2 in ATDC5 cells was measured by Western blotting. (B) and (D) show the densitometric analysis of the immunoblot was expressed as the percentage of control, *P < 0.05, **P < 0.01 vs control groups, The results represent prototypical examples of experiments replicated at least three times.

### 3.3 Effect of NPW‐23 on ATDC5 proliferation

As NPW and NPB were thought to act as regulators of cell proliferation, we studied the effect of NPW‐23 on ATDC5 proliferation. The cell proliferation assays showed that NPW‐23 (200 ng/mL) stimulated ATDC5 proliferation by more than 30% compared with the control cultures (Figures 3, 4, 5, 6A‐B). CYM 50769 (at either 1 or 3 μmol/L) is an antagonist of GPR7, which attenuated the NPW‐23‐induced cell proliferation in a dose‐dependent manner (Figure 3C). We transfected ATDC5 cells with specific siRNA to down‐regulate the GPR7 in order to further study the roles of GPR7 in the effect of NPW‐23. The results showed that knocking down the GPR7 decreased the cell viability that was induced by the NPW‐23(Figures 3, 4, 5, 6D‐E). These results further support that NPW‐23 stimulates the proliferation of ATDC5 by mediating GPR7. In order to investigate the mechanisms by which the NPW‐23 pathway exerts its action on proliferation in the ATDC5 cells, cells were exposed to H‐89 (the PKA pathway inhibitor, 1 μmol/L), Chele (the PKC pathway inhibitor, 3 μmol/L), PD98059 (the ERK1/2 pathway inhibitor, 30 μmol/L), JNK inhibitor (the JNK1/2 MAPK inhibitor, 10 μmol/L) and SB203580 (the p38 MAPK inhibitor, 3 μmol/L), respectively. Our results showed that H‐89, Chele and PD98059 reversed the cell viability of the stimulated NPW‐23, but the JNK inhibitor and PD169316 had no effect (Figure 3F).

### 3.4 Effect of NPW‐23 on markers of chondrogenic differentiation

To investigate the effects of NPW‐23 on the chondrogenic differentiation, ATDC5 cells were cultured in differentiation medium (1% ITS) and incubated with or without NPW‐23 (200 ng/mL) up to 21 days, then the cells were stained using Alizarin red and Alcian blue, which are key indicators of chondrocytic metabolism and mineralization, respectively. Consistent with the previous studies, we observed that the blue and red colours both increased in a time‐dependent manner (Figures 4, 5, 6A‐B**)**.The Alcian blue stain indicated that the proteoglycan synthesis was strongly stimulated by the NPW‐23 on days 7, 14 and 21 (Figure 4A).However, the Alizarin red stain showed no effect on mineralization following NPW‐23 treatment when compared with the control groups (Figure 4B).

We further investigated the effects of NPW‐23 on the expression of chondrogeneis markers in the ATDC5 cells, cells were cultured in differentiation medium (1% ITS) and were incubated with or without NPW‐23 (200 ng/mL) for a certain number of days. It has been reported that induction of the cyclin‐dependent kinase inhibitorp21^Cip‐1/SDI‐1/WAF‐1 ^(p21) is essential for the chondrogenic differentiation of ATDC5 cells.^22^ The results from the real‐time PCR showed that increased expression of p21 in the NPW‐23 treatment group vs the control groups on days 7, 14 and 21 (Figure 4C). Hence, NPW‐23 could promote the proliferation of ATDC5 cells into chondrocytes by stimulating the expression of p21, which is the main feature of proliferating chondrocytes. Coll II was expressed on day 4 and the expression increased with time on days 7 and 14, then switching off during the late‐phase of ATDC5 differentiation on day 21. Meanwhile, the NPW‐23 treatment significantly increased Coll II expression on days 7 and 14 (Figure 4D). Aggrecan, the proteoglycan, was mainly expressed in the ATDC5 cells in the control group on day 4. This expressing increased until day 14 in the culture. Then, it decreased significantly on day 21, and the expression of aggrecan increased approximately 1.8‐ and 2.15‐fold when stimulated by NPW‐23 on days 7 and day 14, respectively (Figure 4E). These data support the findings from the Alcian blue staining, which showed that NPW‐23 treatment increases Alcian blue staining because aggrecan is one of the major contributors to proteoglycan staining. NPW‐23 significantly increased the expression of Sox‐9 in the culture on days 7, 14 and 21 (Figure 4F**)**, but the expression of Coll X and Runx2 were not affected by NPW‐23 and were even up‐regulated during ATDC5 differentiation (Figure 4G‐H). We further analysed the effect of NPW‐23 on the ALP activity. As shown in Figure 4I, the activity of the ALP did not increase significantly during the late stage of differentiation, thus emphasizing the limitations of the in vitro systems.

### 3.5 The effect of NPW‐23 on major regulatory signalling pathways in chondrocytes

We used RT‐PCR to study the major regulators of chondrogenesis that mediate the effects of NPW‐23ʹ on ATDC5 cells. As shown in Figure 5A, the NPW‐23 increased early differentiation when evaluated by Coll II mRNA expression, which was sensitive to the specific inhibitors of the PKA, PKC and p38 MAPK pathways, while the ERK1/2 and JNK inhibitors had no effect. The aggrecan gene expression, which acts as a marker of early chondrocyte differentiation, showed similar results (Figure 5B). Furthermore, p21 expression, which is essential for the chondrogenesis of ATDC5 cells, was inhibited by the specific inhibitors of PKA, PKC, ERK1/2 and p38 MAPK, but showed no reaction to the JNK inhibitor(Figure 5C). Taken together, these data show that the PKA, PKC, p38 MAPK and ERK1/2 signalling pathways mediate NPW‐23, which increases the early differentiation of ATDC5 cells, but the JNK1/2 pathway is not related to this process. Furthermore, we examined the effect of NPW‐23 on the phosphorylation of kinases through Western blotting. NPW‐23 time‐ and dose‐dependent enhanced the phosphorylation level of PKA, PKC, p38 MAPK and ERK1/2. The NPW‐23 (200 ng/mL) increased the phosphorylation level within 20 minutes and reached the peak level at 40‐80 minutes (Figure 6A‐D).

## 4 DISCUSSION

The G protein‐coupled receptor (GPR) plays a key role in regulating bone physiology and pathology. As one subtype of the GPR family, GPR7 is expressed in many brain and periphery tissues.^25^ Neuropeptides W is a natural agonist of GPR7 and has universal distribution in the brain tissue.^3^, ^7^ It is also expressed in periphery tissues and is involved in different cellular processes via GPR7, including hormonal and energy homeostasis.^27^, ^28^, ^29^ Our results showed the first time that NPW and GPR7 are expressed in the ATDC5 cells and growth plates of the mice. The process of endochondral bone is regulated by chondrocytes in the growth plate; therefore, our immunohistochemical results showed higher levels of NPW and GPR7 in the proliferative chondrocytes in the lower zone of the growth plate, which indicates that NPW is possibly secreted by proliferative chondrocytes in order to regulate chondrocyte proliferation through GPR7.We also found that ATDC5 proliferation was strong stimulated by NPW‐23 and inhibited by CYM50769 (GPR7 antagonist) and siRNA for GPR7, which suggests that NPW‐23 may act through GPR7 to regulate the proliferation of ATDC5 cells. This agrees with previous studies that show NPW can regulate cell proliferation from cultured rat adrenocortical cells or calvarial osteoblast‐like cells. This study shows for the first time that the NPW/GPR7 system promotes chondrocyte proliferation.

ITS treatment in ATDC5 cells is an effective model for studying chondrocyte differentiation.^30^ Our results show that NPW‐23 expression increased on day 4, peaked on day 7 and decreased on day 14. In contrast, the GPR7 mRNA decreased in a time‐dependent manner during the differentiation of the ATDC5 cells, which was induced by ITS. These results show that NPW‐active GPR7 may regulate the early differentiation of ATDC5.

The expression of mRNA in downstream signalling pathways was tested to study the mechanism of NPW that is involved in chondrogenesis. The p21 is a well‐known G1 cell cycle regulator that is related through chondrocyte proliferation and differentiation. Furthermore, the increase of endogenous p21 stimulated early chondrogenic differentiation in the ATDC5 cells.^22^ Sox9, a chondrogenic transcription factor, is necessary for chondrogenesis.^31^ In this study, the expression of Sox‐9 increased on day 14 during the differentiation of the ATDC5 cells, which was induced by ITS. Neuropeptides W induced dramatic up‐regulation of p21 and Sox‐9 signalling molecules, which supported the finding that NPW promotes the proliferation of ATDC5 through the interaction effect during bone development.^11^, ^32^ We also found that NPW induced differentiation of ATDC5, which coincided with augmented Coll II and aggrecan expression. Both Coll II and aggrecan are known to play a vital role during early‐stage chondrogenesis. While NPW‐23 did not increase the activity of ALP in ATDC5 during differentiation, the induction of ALP activity in chondrocytes correlates with the accumulation and mineralization of matrix vesicles in ECM during the late stage of differentiation.^33^, ^34^ These results indicate that NPW‐23 contributes to ATDC5 early‐stage chondrogenesis, but not late‐phase chondrogenic differentiation.

All sorts of kinases are relevant to the extracellular chondro‐regulatory molecules in the nuclear transcription factors, including PKA and PKC.^13^ PKC play an important role in many signal‐transducing kinases that promote chondrocyte proliferation and differentiation.^35^, ^36^ As PKA and PKC cascades are involved indifferent cellular processes of NPB/NPW, we investigated the related signalling pathway of NPW action in chondrocytes.^9^, ^29^ In the current research, H‐89 (PKA inhibitor) and Chele (PKC inhibitor) inhibited the expression of Coll II mRNA, which was induced by NPW‐23. Protein kinase A is known to phosphorylate Sox9 and enhance its transcriptional activity.^37^ It has been reported that PKA regulates chondrogenesis in chick limb bud mesenchymal cells via a PKCα‐dependent manner.^38^ Protein kinase C is a driver of multiple signal transducing kinases, including ERK1/2 and p38 MAPK, which promote the proliferation and differentiation of chondrocytes.^13^ The major MAPK subtypes, including ERK1/2, JNK and p38MAPK,^39^ are known to participate in chondrogenic differentiation.^40^ ERK1/2 promotes cartilage formation by promoting early differentiation, p38 MAPK stimulates cartilage formation by acting on early and late cultures, and JNK acts as a negative regulator of cartilage formation. SB203580 (p38 inhibitor) and PD98059 (ERK1/2 inhibitor) reduce the mRNA expression of p21 that is induced by NPW‐23. These results confirm reports that the ERK1/2 and p38 MAPK pathways promote the expression of p21 in ATDC5 cells.^32^ Interestingly, H89, Chele and SB203580 abrogate the NPW‐23‐induced Coll II and aggrecan mRNA, while PD98059 has none of these effects. These results are in line with previous studies, which show that ERK1/2 is the main pathway stimulated by proliferation, and the p38 MAPK pathway mainly regulates early differentiation.^36^, ^40^ Moreover, NPW‐23 stimulated the phosphrylation of PKA, PKC and p38 in a time‐ and concentration‐dependent manner. These data imply that the promotion of proliferation and differentiation of ATDC5 cells induced by NPW is mediated by the PKA and PKC signalling pathways. It may be true that PKC mediates the p38 MAPK signal pathway in ATDC5 cells.^13^ However, further investigation is required to determine the contribution of PKA/PKC signalling in mediating GPR7 activation in murine chondrocyte.

In conclusion, this study is the first to suggest that NPW binds GPR7 in order to promote proliferation and early differentiation of murine chondrocyte. This effect was because of the activation of the adenylate cyclase/PKA and the phospholipase C/PKC cascades, as well as its promoted p38 MAPK phosphorylation. Earlier studies also suggest that NPW/NPB exists in the blood and systemic veins of rats,^41^ which indicates that NPW/GPR7 also affects cartilage functions in vivo. However, we failed to identify the possible mechanism of NPW action on chondrocyte in vivo. We hope to study this in the future.

## CONFLICT OF INTEREST

The authors declare no conflicts of interest.

## References

[1] Fujii R , Yoshida H , Fukusumi S , et al. Identification of a neuropeptide modified with bromine as an endogenous ligand for GPR7. J Biol Chem. 2002;277:34010‐34016. 12118011 10.1074/jbc.M205883200

[2] Shimomura Y , Harada M , Goto M , et al. Identification of neuropeptide W as the endogenous ligand for orphan G‐protein‐coupled receptors GPR7 and GPR8. J Biol Chem. 2002;277:35826‐35832. 12130646 10.1074/jbc.M205337200

[3] Lee DK , Nguyen T , Porter CA , Cheng R , George SR , O'Dowd BF . Two related G protein‐coupled receptors: the distribution of GPR7 in rat brain and the absence of GPR8 in rodents. Brain Res Mol Brain Res. 1999;71:96‐103. 10407191 10.1016/s0169-328x(99)00171-0

[4] Takenoya F , Kageyama H , Shiba K , Date Y , Nakazato M , Shioda S . Neuropeptide W: a key player in the homeostatic regulation of feeding and energy metabolism? Ann NY Acad Sci. 2010;1200:162‐169. 20633144 10.1111/j.1749-6632.2010.05642.x

[5] Sakurai T . NPBWR1 and NPBWR2: implications in energy homeostasis, pain, and emotion. Frontiers in endocrinology. 2013;4:23. 23515889 10.3389/fendo.2013.00023 PMC3600615

[6] Zaratin PF , Quattrini A , Previtali SC , Comi G , Hervieu G , Scheideler MA . Schwann cell overexpression of the GPR7 receptor in inflammatory and painful neuropathies. Mol Cell Neurosci. 2005;28:55‐63. 15607941 10.1016/j.mcn.2004.08.010

[7] Brezillon S , Lannoy V , Franssen JD , et al. Identification of natural ligands for the orphan G protein‐coupled receptors GPR7 and GPR8. J Biol Chem. 2003;278:776‐783. 12401809 10.1074/jbc.M206396200

[8] Bu G , Lin D , Cui L , et al. Characterization of neuropeptide B (NPB), neuropeptide W (NPW), and their receptors in chickens: evidence for NPW being a novel inhibitor of pituitary GH and prolactin secretion. Endocrinology. 2016;157:3562‐3576. 27399877 10.1210/en.2016-1141

[9] Mazzocchi G , Rebuffat P , Ziolkowska A , Rossi GP , Malendowicz LK , Nussdorfer GG . G protein receptors 7 and 8 are expressed in human adrenocortical cells, and their endogenous ligands neuropeptides B and W enhance cortisol secretion by activating adenylate cyclase‐ and phospholipase C‐dependent signaling cascades. J Clin Endocrinol Metab. 2005;90:3466‐3471. 15797961 10.1210/jc.2004-2132

[10] Stork PJ , Schmitt JM . Crosstalk between cAMP and MAP kinase signaling in the regulation of cell proliferation. Trends Cell Biol. 2002;12:258‐266. 12074885 10.1016/s0962-8924(02)02294-8

[11] Juhasz T , Matta C , Somogyi C , et al. Mechanical loading stimulates chondrogenesis via the PKA/CREB‐Sox9 and PP2A pathways in chicken micromass cultures. Cell Signal. 2014;26:468‐482. 24333667 10.1016/j.cellsig.2013.12.001

[12] Ciarmatori S , Kiepe D , Haarmann A , Huegel U , Tonshoff B . Signaling mechanisms leading to regulation of proliferation and differentiation of the mesenchymal chondrogenic cell line RCJ3.1C5.18 in response to IGF‐I. J Mol Endocrinol. 2007;38:493‐508. 17446238 10.1677/jme.1.02179

[13] Yosimichi G , Kubota S , Nishida T , et al. Roles of PKC, PI3K and JNK in multiple transduction of CCN2/CTGF signals in chondrocytes. Bone. 2006;38:853‐863. 16431170 10.1016/j.bone.2005.11.016

[14] Andreis PG , Rucinski M , Neri G , et al. Neuropeptides B and W enhance the growth of human adrenocortical carcinoma‐derived NCI‐H295 cells by exerting MAPK p42/p44‐mediated proliferogenic and antiapoptotic effects. Int J Mol Med. 2005;16:1021‐1028. 16273281

[15] Hochol A , Albertin G , Nussdorfer GG , et al. Effects of neuropeptides B and W on the secretion and growth of rat adrenocortical cells. Int J Mol Med. 2004;14:843‐847. 15492854

[16] Niedermair T , Kuhn V , Doranehgard F , et al. Absence of substance P and the sympathetic nervous system impact on bone structure and chondrocyte differentiation in an adult model of endochondral ossification. Matrix Biol. 2014;38:22‐35. 25063231 10.1016/j.matbio.2014.06.007

[17] Saito H , Nakamachi T , Inoue K , et al. Autocrine effects of neuromedin B stimulate the proliferation of rat primary osteoblasts. J Endocrinol. 2013;217:141‐150. 23428580 10.1530/JOE-12-0488

[18] Cornish J , Callon KE , Mountjoy KG , et al. alpha ‐melanocyte‐stimulating hormone is a novel regulator of bone. Am J Physiol Endocrinol Metab. 2003;284:E1181‐E1190. 12618359 10.1152/ajpendo.00412.2002

[19] Ziolkowska A , Rucinski M , Tyczewska M , Malendowicz LK . Neuropeptide B (NPB) and neuropeptide W (NPW) system in cultured rat calvarial osteoblast‐like (ROB) cells: NPW and NPB inhibit proliferative activity of ROB cells. Int J Mol Med. 2009;24:781‐787. 19885618 10.3892/ijmm_00000292

[20] Yao Y , Wang Y . ATDC5: an excellent in vitro model cell line for skeletal development. J Cell Biochem. 2013;114:1223‐1229. 23192741 10.1002/jcb.24467

[21] Atsumi T , Miwa Y , Kimata K , Ikawa Y . A chondrogenic cell line derived from a differentiating culture of AT805 teratocarcinoma cells. Cell Differ Dev. 1990;30:109‐116. 2201423 10.1016/0922-3371(90)90079-c

[22] Negishi Y , Ui N , Nakajima M , et al. p21Cip‐1/SDI‐1/WAF‐1 gene is involved in chondrogenic differentiation of ATDC5 cells in vitro. J Biol Chem. 2001;276:33249‐33256. 11406616 10.1074/jbc.M010127200

[23] Wang R , Yan F , Liao R , Wan P , Little PJ , Zheng W . Role of brain‐derived neurotrophic factor and nerve growth factor in the regulation of Neuropeptide W in vitro and in vivo. Mol Cell Endocrinol. 2017;447:71‐78. 28249734 10.1016/j.mce.2017.02.040

[24] Shen W , Wang L , Pi R , Li Z , Rikang W . L‐F001, a multifunctional ROCK inhibitor prevents paraquat‐induced cell death through attenuating ER stress and mitochondrial dysfunction in PC12 cells. Biochem Biophys Res Comm. 2015;464:794‐799. 26187664 10.1016/j.bbrc.2015.07.035

[25] Mediero A , Cronstein BN . Adenosine and bone metabolism. Trends Endocrinol Metab. 2013;24:290‐300. 23499155 10.1016/j.tem.2013.02.001 PMC3669669

[26] Chottova DM . Distribution and function of neuropeptides W/B signaling system. Front physiol. 2018;9:981. 30087623 10.3389/fphys.2018.00981 PMC6067035

[27] Skrzypski M , Pruszynska‐Oszmalek E , Rucinski M , et al. Neuropeptide B and W regulate leptin and resistin secretion, and stimulate lipolysis in isolated rat adipocytes. Regul Pept. 2012;176:51‐56. 22484289 10.1016/j.regpep.2012.03.004

[28] Li H , Kentish SJ , Kritas S , et al. Modulation of murine gastric vagal afferent mechanosensitivity by neuropeptide W. Acta Physiol. 2013;209:179‐191. 10.1111/apha.12154 23927541

[29] Ji L , Zhu H , Chen H , et al. Modulation of CaV1.2 calcium channel by neuropeptide W regulates vascular myogenic tone via G protein‐coupled receptor 7. J Hypertens. 2015;33:2431‐2442. 26536090 10.1097/HJH.0000000000000723

[30] Challa TD , Rais Y , Ornan EM . Effect of adiponectin on ATDC5 proliferation, differentiation and signaling pathways. Mol Cell Endocrinol. 2010;323:282‐291. 20380870 10.1016/j.mce.2010.03.025

[31] Lee JS , Im GI . SOX trio decrease in the articular cartilage with the advancement of osteoarthritis. Connect Tissue Res. 2011;52:496‐502. 21728837 10.3109/03008207.2011.585409

[32] Nakajima M , Negishi Y , Tanaka H , Kawashima K . p21(Cip‐1/SDI‐1/WAF‐1) expression via the mitogen‐activated protein kinase signaling pathway in insulin‐induced chondrogenic differentiation of ATDC5 cells. Biochem Biophys Res Comm. 2004;320:1069‐1075. 15249198 10.1016/j.bbrc.2004.06.057

[33] Shukunami C , Ishizeki K , Atsumi T , Ohta Y , Suzuki F , Hiraki Y . Cellular hypertrophy and calcification of embryonal carcinoma‐derived chondrogenic cell line ATDC5 in vitro. J Bone Miner Res. 1997;12:1174‐1188. 9258747 10.1359/jbmr.1997.12.8.1174

[34] Shukunami C , Ohta Y , Sakuda M , Hiraki Y . Sequential progression of the differentiation program by bone morphogenetic protein‐2 in chondrogenic cell line ATDC5. Exp Cell Res. 1998;241:1‐11. 9633508 10.1006/excr.1998.4045

[35] Choi B , Chun JS , Lee YS , Sonn JK , Kang SS . Expression of protein kinase C isozymes that are required for chondrogenesis of chick limb bud mesenchymal cells. Biochem Biophys Res Comm. 1995;216:1034‐1040. 7488176 10.1006/bbrc.1995.2724

[36] Chang SH , Oh CD , Yang MS , et al. Protein kinase C regulates chondrogenesis of mesenchymes via mitogen‐activated protein kinase signaling. J Biol Chem. 1998;273:19213‐19219. 9668109 10.1074/jbc.273.30.19213

[37] Huang W , Zhou X , Lefebvre V , de Crombrugghe B . Phosphorylation of SOX9 by cyclic AMP‐dependent protein kinase A enhances SOX9's ability to transactivate a Col2a1 chondrocyte‐specific enhancer. Mol Cell Biol. 2000;20:4149‐4158. 10805756 10.1128/mcb.20.11.4149-4158.2000 PMC85784

[38] Yoon YM , Oh CD , Kang SS , Chun JS . Protein kinase A regulates chondrogenesis of mesenchymal cells at the post‐precartilage condensation stage via protein kinase C‐alpha signaling. J Bone Miner Res. 2000;15:2197‐2205. 11092400 10.1359/jbmr.2000.15.11.2197

[39] Wang R , Yang J , Peng L , et al. Gardenamide A attenuated cell apoptosis induced by serum deprivation insult via the ERK1/2 and PI3K/AKT signaling pathways. Neuroscience. 2015;286:242‐250. 25485482 10.1016/j.neuroscience.2014.11.056

[40] Bobick BE , Kulyk WM . Regulation of cartilage formation and maturation by mitogen‐activated protein kinase signaling. Birth Defects Res C Embryo Today. 2008;84:131‐154. 18546337 10.1002/bdrc.20126

[41] Mondal MS , Yamaguchi H , Date Y , et al. Neuropeptide W is present in antral G cells of rat, mouse, and human stomach. J Endocrinol. 2006;188:49‐57. 16394174 10.1677/joe.1.06195

